# FUNCTIONAL WELLNESS AMONG OLDER ADULTS WITH HIV/AIDS: BARRIERS AND FACILITATORS TO HEALTH AND LONGEVITY

**DOI:** 10.1093/geroni/igad104.1346

**Published:** 2023-12-21

**Authors:** Erin Robinson, Molly Perkins, Mark Brennan-Ing

## Abstract

In the United States, older adults make up over half of all people living with HIV/AIDS (PLWH). Today, this population is living longer and healthier lives due to advancements in HIV treatment. However, older adults living with HIV (OALWH) present a unique set of challenges as they face aging-related health considerations that intersect with their HIV status. This symposium brings together leading researchers in the field of HIV and aging, to highlight the complexity of this topic and present innovative research that examines the overall health and well-being of OALWH. Our first presentation is a qualitative inquiry into barriers to successful aging that are shared between older women and men living with HIV/AIDS, as well as differences. Our second presentation used a latent health profile model to identify subgroups of PLWH who had been discharged from a short-term skilled nursing facility (SNF). The goal of this research was to identify risk for institutional admission following their SNF stay. Our third presentation examined age-varying cognitive impairment among a sample of PLWH, people who use methamphetamines, and meth-negative/seronegative individuals to determine whether social relationships meditate the complex associations between meth use and cognitive impairment for PLWH and without HIV. And finally, our fourth presentation examined perspectives on weight change and body image among PLWH and the extent to which age and gender have an impact on those attitudes. Findings from this symposium demonstrates the complexity of what it means to be an OALWH and the various considerations for their well-being. This is a HIV, AIDS and Older Adults Interest Group Sponsored Symposium.

